# Metabolic engineering of ketocarotenoids biosynthetic pathway in *Chlamydomonas reinhardtii* strain CC-4102

**DOI:** 10.1038/s41598-020-67756-2

**Published:** 2020-07-01

**Authors:** Nam Trung Tran, Ralf Kaldenhoff

**Affiliations:** Department of Biology, Applied Plant Sciences, Technische Universität Darmstadt, Schnittspahn Strasse 10, 64287 Darmstadt, Germany

**Keywords:** Plant biotechnology, Secondary metabolism

## Abstract

In *Chlamydomonas reinhardtii*, ketocarotenoid biosynthesis is limited to the diploid zygospore stage. In this study, we attempted to engineer the ketocarotenoid pathway into *Chlamydomonas* haploid vegetative green cells by overexpressing the key enzyme ß-carotene ketolase (CrBKT). We chose strain CC-4102 for the approach; competitive pathways, α-carotene biosynthesis and xanthophyll cycle are silenced in this strain. Driven by the strong constitutive HSP70/RBCS2 promoter CrBKT overexpression resulted in the production of canthaxanthin, the ketolation product from ß-carotene as well as a drastic reduction in the chlorophyll concentration. Intriguingly, these phenotypes could only be detected from lines transformed and grown heterotrophically in the dark. Once exposed to light, these transformants lost the aforementioned phenotypes as well as their antibiotic resistance. This phenomenon is in agreement with the fact that we were unable to recover any canthaxanthin-producing line among light-selected transformants.

## Introduction

Ketocarotenoids are a special group of carotenoids characterized by the presence of one or several carbonyl groups in their ß-ionone rings. The electron-withdrawing effect of keto moieties on the carotenoid’s conjugated π-system results in a slight shift of absorption maximum from 450 nm (yellow) to 470 nm (red). Thus, ketocarotenoids are easily recognizable by their red hue. Due to their excellent anti-oxidative characteristics, ketocarotenoids such as astaxanthin and canthaxanthin rank among the highest-value products on the carotenoid pigment market^1^. While market demands are still being met mainly by the chemical industry, the interests in ketocarotenoids produced in biological systems has witnessed skyrocketing growth in the recent years^2^.

In contrast to non-ketolated carotenoids such as ß-carotene, lutein, zeaxanthin, violaxanthin etc., all of which are ubiquitous among photosynthetic organisms, the biosynthesis of ketocarotenoids is limited to a small groups of organisms including several marine bacteria^3^, the fungus *Xanthophyllomyces dendrorhous*^4^, microalgae^5^ and flowers of *Adonis aestivalis*^6^. Most prominent among these species is the unicellular green alga *Haematococcus pluvialis*, which can accumulate astaxanthin at very high levels up to 7% of its dried weight^7^. Huge algal farms growing *Haematococcus* for astaxanthin extraction have been established in several countries including the USA, Israel and China^8^. Industrial production of astaxanthin in *Haematococcus*, however is hampered by several limiting factors such as the alga’s slow growth rate and low cell density, high risk of contamination^9^, parasitic disease^10^, high energy cost for induction of carotenogenesis (requiring high light intensity) and difficulty breaking the thick cell wall of haematocysts^11^. Thus, there is a great and obvious need to develope new, alternative biological platforms for ketocarotenoids production.

Significant efforts have been made to engineer the ketocarotenoid biosynthetic pathway (especially of the high-value astaxanthin) into already existing carotenoid biosynthesis of model organisms such as bacteria^12^, cyanobacteria^13^, yeast^14^, higher plants^15–17^ or microalgae^18–20^. The experimental strategy often involves the introduction of the key enzyme, ß-carotene ketolase (BKT) derived from an astaxanthin-producing organism, into the host’s cells. The results of these approaches vary from complete reddening of plant tissues due to pigment accumulation to only trace amounts of detected ketocarotenoids. Due to the promiscuous nature of the enzyme, a large number of intermediates or side-products are accumulated in conjunction with the target ketocarotenoid^21^. The dearth of isoprenoid precursors and metabolic sink prevent the maximal efficiency of carotenoid biosynthesis^22^. Spatial separation of enzyme–substrate is another factor that might result in low production of ketocarotenoids^23,24^. Feedback-inhibition of carotenogenesis due to its own excessive end-product has also been reported^25^. Overall, these studies reflect the complexity of ketocarotenoid biosynthesis and the fact that in many cases introducing a foreign protein in a metabolic network only represents the first step of metabolic engineering.

For at least 70 years, the unicellular green alga *Chlamydomonas reinhardtii* has served as a model organism for research on photosynthesis, flagellate structure and function, chloroplast biogenesis, light perception, cell–cell recognition and cell cycle control, among many other biological processes^26^. *Chlamydomonas* also belongs to a limited number of microalgae whose transformation has been routinely achieved by thoroughly developed molecular toolkits^27^. It was long assumed that *Chlamydomonas reinhardtii* does not accumulate ketocarotenoid. This assumption was disputed in 2011 when large amounts of ketocarotenoids, including 4-ketolutein, 4-ketolutein esters, astaxanthin, astaxanthin esters and canthaxanthin, were detected in *Chlamydomonas* diploid zygospores^28^. A ß-carotene ketolase variant, termed CrBKT, was identified earlier from sequencing data of *Chlamydomonas* genome^29^. Functional analysis in *E. coli* showed that CrBKT is a diketolase (i.e. able to add carbonyl groups to both ß-ionone rings) capable of converting ß-carotene to canthaxanthin and zeaxanthin to astaxanthin with high efficiency^12^. CrBKT is highly expressed in *Chlamydomonas* zygospores, while in vegetative cells its expression is kept at minimal level. This pattern explains the lack of ketocarotenoids in these cells. CrBKT has been successfully utilized to introduce astaxanthin production into *Arabidopsis*^12^, tomato^16^, tobacco^15^ and rice^17^ among other species.

While commercial production of ketocarotenoid in *Chlamydomonas* zygospores is not economical (the zygospore maturation alone takes at least one month!), vegetative *Chlamydomonas* cells have shown a great potential as host for production of a wide range of high-value compounds and biotechnological products^30^. In this paper, we report about our approach to engineer the ketocarotenoid biosynthetic pathway into *Chlamydomonas* vegetative cells by overexpressing its native enzyme CrBKT. The resulting transformants accumulated canthaxanthin and also displayed significant changes in chlorophyll content. These findings indicate a cross-talk mechanism between ketocarotenoid- and chlorophyll biosynthesis.

## Results

### Selection of *Chlamydomonas* strain for ketocarotenoid metabolic engineering

**Figure 1:**
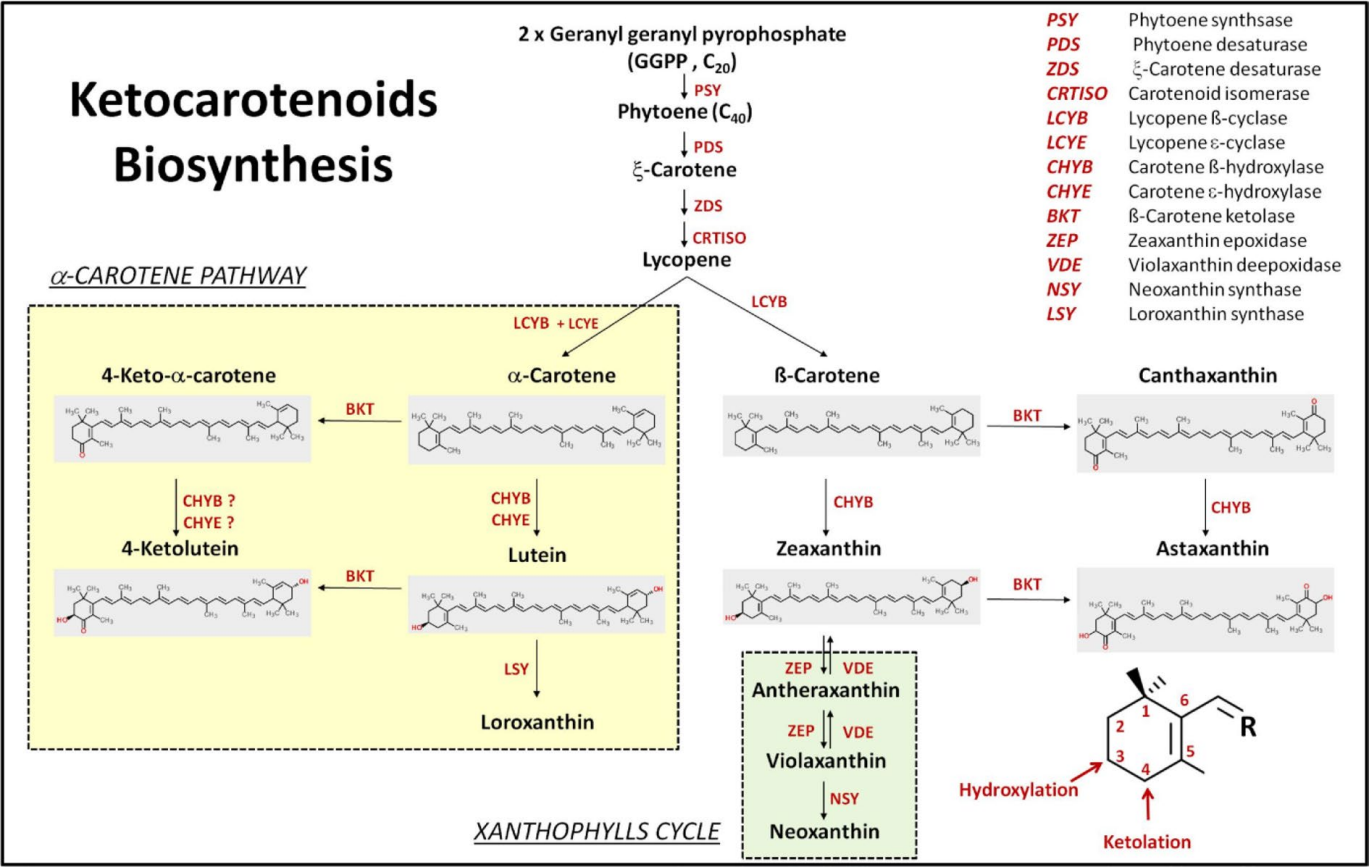
Biosynthetic pathways of carotenoids and ketocarotenoids in *Chlamydomonas reinhardtii*. In the lower right corner is the numbering of ß-ionone ring as well as positions where hydroxylation and ketolation reactions take place. In order to direct cell’s resources towards biosynthesis of highly valuable canthaxanthin and astaxanthin, we identified two competing side pathways—the α-carotene biosynthesis and xanthophylls cycle—that need to be eliminated. These pathways are highlighted in coloured boxes.

Studies in *Haematococcus pluvialis*^31^, *Chromochloris zofingiensis*^5^, *Agrobacterium aurantiacum*^3^, *Xanthophyllomyces dendrorhous*^32^ and *Adonis aestivalis*^6^ have indicated that ketocarotenoids are synthesized from carotenoids. Two types of reactions are usually involved: ketolation (i.e. addition of the carbonyl groups to the ß-ionone rings) catalyzed by ß-carotene ketolases (BKT/CrtW/CrtO) and hydroxylation (i.e. addition of the hydroxyl groups to the ß-ionone rings) catalyzed by ß-carotene hydroxylases (CHYb/CrtY/CrtR; Fig. 1). Like many other secondary metabolic processes, the enzymes of ketocarotenoid biosynthesis are promiscuous: They can accept a wide variety of substrates. For example, CrBKT can process ß-carotene to echinenone, echinenone to canthaxanthin, zeaxanthin to adonixanthin, adonixanthin to astaxanthin, α-carotene to 4-keto-α-carotene and lutein to 4-ketolutein^33^ .

To streamline resources towards the biosynthesis of highly valuable ketocarotenoids—canthaxanthin or astaxanthin—competitive metabolic side pathways can be silenced. As depicted in Fig. 1, such pathways include the biosynthesis of α-carotenoids and those of xanthophylls cycle pigments (violaxanthin, antheraxanthin and neoxanthin). Silencing these pathways requires disruption of the conversion steps of lycopene to α-carotene and of zeaxanthin to violaxanthin. In *Chlamydomonas reinhardtii*, there have been successful reports of CRISPR-Cas9- and RNA interference (RNAi)-mediated gene knock-out/knock-down^34–37^ but these techniques have not been well established.

*Chlamydomonas* strain CC-4102 (genotype *npq2-2 npq1 lor1)*, in which both the α-carotenoids biosynthesis and the xanthophylls cycle are defective, has been previously isolated and characterized^38^. This strain is characterized by reduced non-photochemical quenching (NPQ), accumulation of zeaxanthin as well as the near absence of lutein, violaxanthin, antheraxanthin and neoxanthin. Surprisingly, even with such defective photoprotective mechanisms, CC-4102 cells can still grow at both low- and high light conditions. These findings suggest that zeaxanthin protects cells from photooxidation. For ketocarotenoids metabolic engineering, strain CC-4102 holds several advantages over the frequently used CC-124 or CC-4350 *Chlamydomonas* strains. The absence of many major carotenoids leads to less clustered chromatograms, making it easier to detect newly formed ketocarotenoids. Their absence also reduces the possibilities of side products – unwanted ketolated substances due to the promiscuous nature of CrBKT. The major carotenoids of CC-4102, ß-carotene and zeaxanthin, are good substrates of CrBKT and the respective products, canthaxanthin and astaxanthin, are high value ketocarotenoids. Consequently, we chose strain CC-4102 for the ketocarotenoid metabolic engineering experiment.

### Transformation and selection of *Chlamydomonas* strain CC-4102

We transformed the algal cells with CrBKT-overexpression vector pChlamy4 CrBKT V5H via electroporation. We selected transformed CC-4102 cells under either mixotroph or heterotroph conditions, as detailed in “Materials and methods” section. In both cases, we obtained numerous zeocin-resistant colonies (184 colonies from mixotrophic selection, 56 colonies from heterotrophic selection: the corresponding transformation efficiencies were 184 and 56 colonies/µg vector DNA, respectively). Transformation with another ble2A bicistronic vector, namely pBR9 mCherry^39^ provided a similar efficiency (186 colonies/µg vector DNA). Most of the CrBKT transformants displayed a dark green color indistinguishable from non-transformed cells. Only on plates cultivated under heterotrophic conditions did we observe four pale green colonies, whose green color seemed much less intense compared to the surrounding dark green ones. We termed such lines DARK-PALE 1–4 and grouped them separately for later analysis.

**Figure 2:**
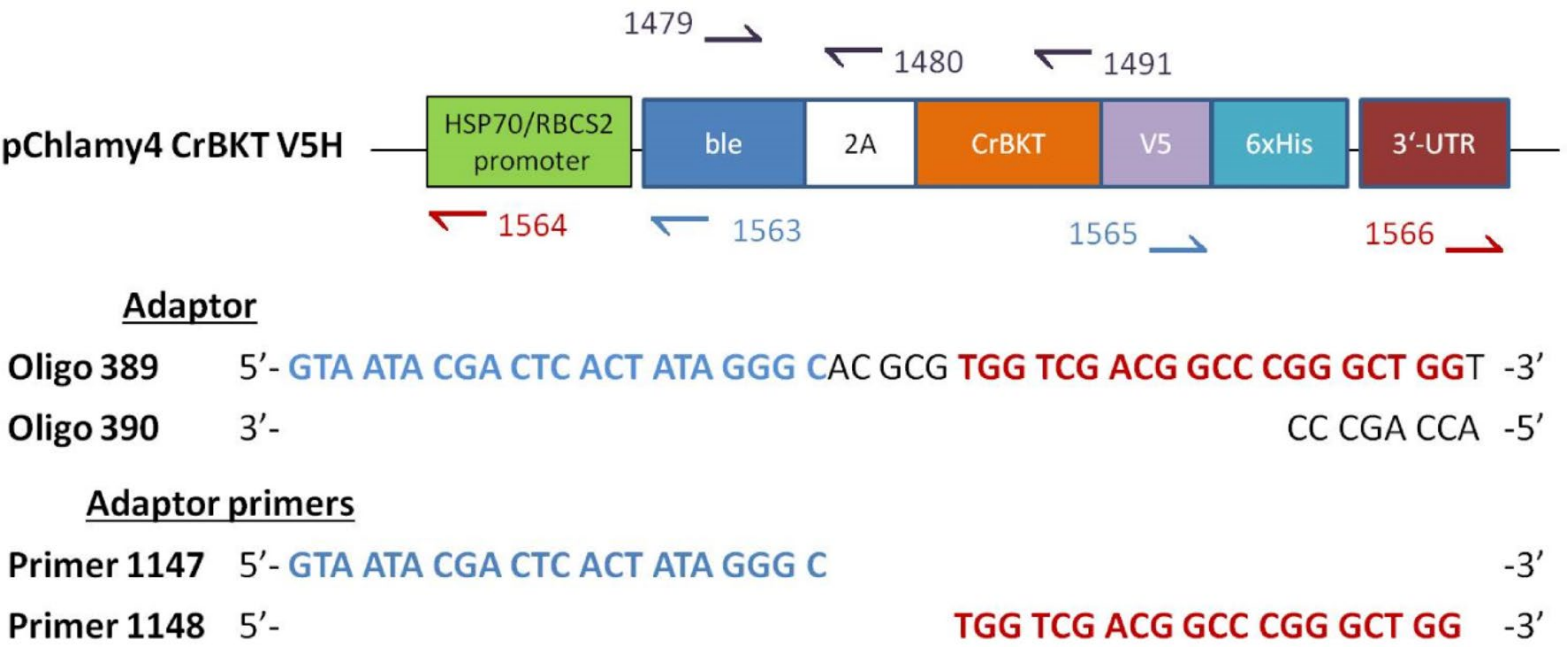
The overexpression vector pChlamy4 CrBKT V5H. Half arrows represent binding positions and 5′-3′ direction of primers used for PCR screening and insertion mapping of transformants. Also shown are the sequences of adaptors and adaptor primers used for insertion mapping. Oligonucleotides 389 and 390 annealed to form asymmetric blunt-ended adaptor. The short strand was extended after first round of PCR.

We extracted genomic DNA from 96 zeocin-resistant lines and performed PCR to confirm the integration of the overexpression construct into genome. We used primers 1479 and 1491, which spanned from the selection marker *ble*, over 2A sequence to the very end of *CrBKT* gene (Fig. 2). Successful integration of the intact *ble2A-CrBKT* resulted in amplification of a 1288 base pair (bp)-long fragment. For comparison, we combined transformants into three groups: dark green lines from mixotrophic selection, dark green lines from heterotrophic selection and pale green lines (named DARK-PALE lines) from heterotrophic selection. As presented in Table 1, the PCR-positive rate among DARK-PALE lines was 100% (4/4), while we observed much lower rates (10% and 5.8%) in other two (dark green) groups.

**Table 1:** Whole cassette amplification (PCR with primers 1479 and 1491).

	Number of colonies	Transformation efficiency	PCR-positive/tested	Percentage (%)
Dark green, mixotrophic	184	184 colonies/µg DNA	4/40	10
Dark green, heterotrophic	52	56 colonies/µg DNA	3/52	5.8
Pale green, heterotrophic (DARK-PALE lines)	4		4/4	100

### Pigment analysis of transformants overexpressing CrBKT

**Figure 3:**
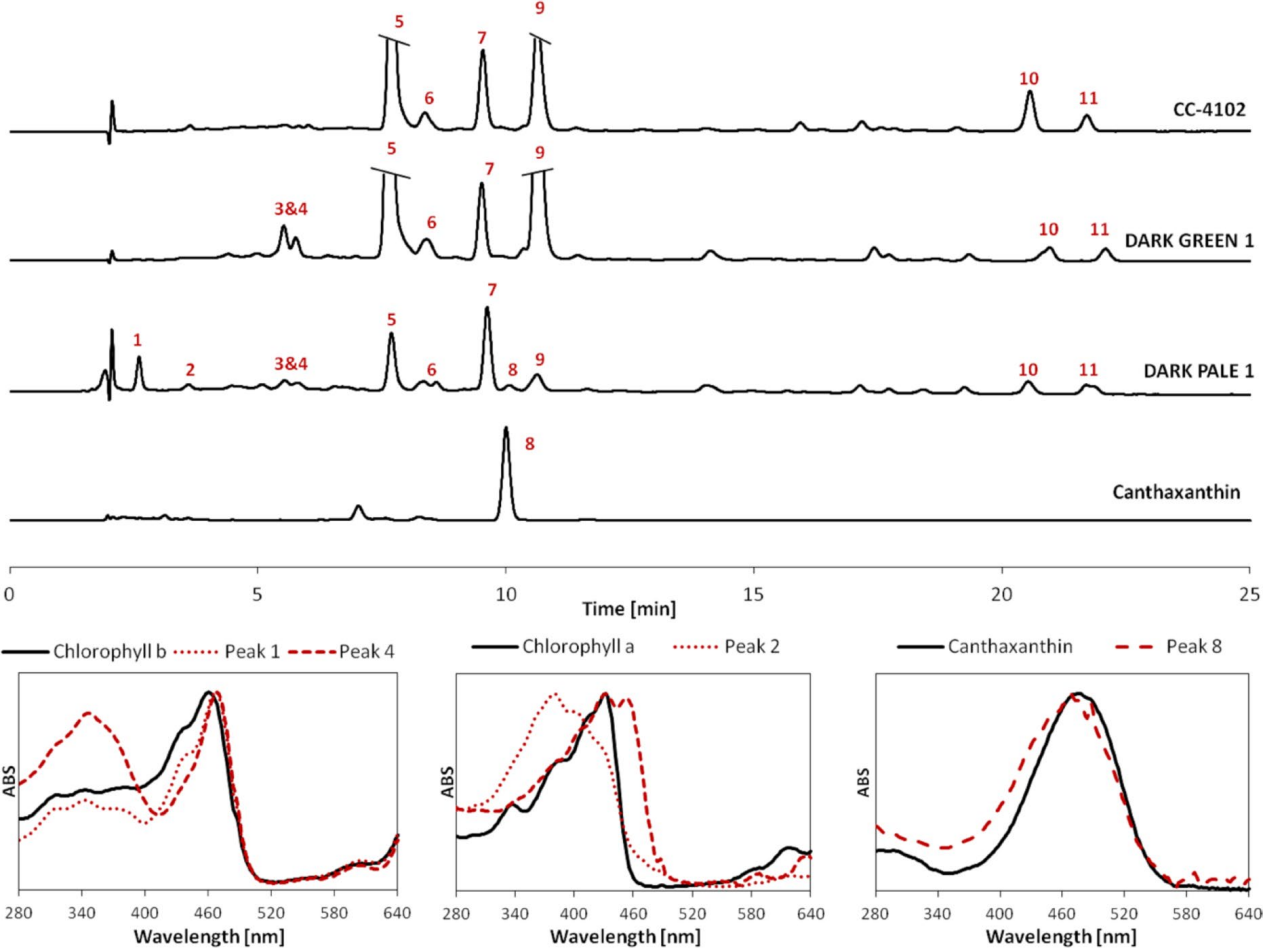
HPLC analysis of pigments extracted from DARK-PALE1 transformant as well as from DARK-GREEN 1 and non-transformed CC-4102 cells. Peaks identification: (1), (2), (3), (4): putative chlorophyll degradation products, (5): chlorophyll *b*, (6): lutein, (7): zeaxanthin, (8): new ketocarotenoid, (9): chlorophyll *a*, (10) and (11): ß-carotene. Below are absorption spectra from 280 to 640 nm of peaks (1), (2), (3), (4), (8), as well of chlorophyll *a*, *b* and canthaxanthin. Peak 8 is identified as canthaxanthin based on identical retention times (peak 8: 10.06 min, canthaxanthin: 10.00 min) and similar absorption spectra.

**Figure 4:**
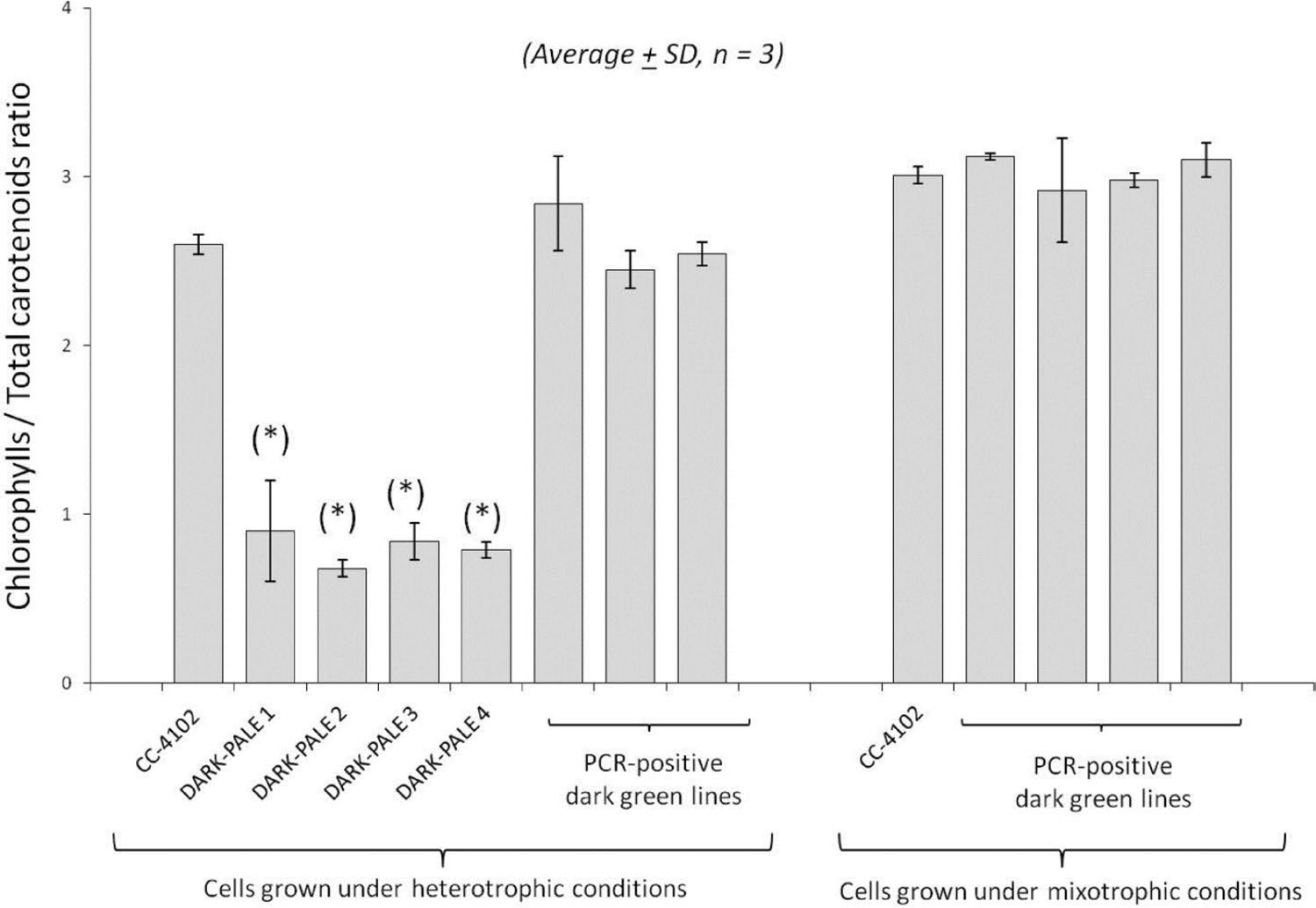
Comparison of chlorophylls/total carotenoids ratios of all PCR-positive CrBKT overepxression transformants. Four DARK-PALE lines display significantly lower Chl/car ratios than both non-transformed CC-4102 cells as well as all dark green lines. (*) denotes statistical significance (*p* < 0.05) compared to non-transformed cells under same growth conditions.

The pigment profiles of all dark green transformants, regardless of whether they were from mixotrophic or heterotrophic selection, were almost identical in pigment profile compared to non-transformed CC-4102 cells under the same growth conditions. On the other hand, there were significant changes among DARK-PALE transformants (Figs. 3, 4). In all DARK-PALE lines, the chlorophyll-to-carotenoid ratio was strongly reduced by a factor of 2 to 3, causing a pale green color. HPLC analysis revealed the presence of canthaxanthin—the diketolation product of ß-carotene in at least two DARK-PALE lines (DARK-PALE 1 and DARK-PALE 4). These data indicatie that in these lines CrBKT was expressed and functional. We estimated canthaxanthin concentrations to be about 10% of total carotenoid concentration (0.1 pg/cell). There was no astaxanthin. We also noticed the presence of many minor peaks whose absorption spectra were similar to those of chlorophyll *a* or chlorophyll *b*. We assume that these minor peaks might be either from degradation of chlorophyll or were accumulated intermediates from chlorophyll biosynthetic pathways.

### Insertion mapping of DARK-PALE transformants

We mapped the 5′- and 3′-flanking regions of DARK-PALE 1 and 3′-flanking region of DARK-PALE 2 (Table S1 and Fig. S2, Supplementary Material). In DARK-PALE 1 5′- and 3′-end mapping yielded different insertion sites in different chromosomes. Such ambiguity was also encountered by Zhang et al*.*^40^ and Pollock et al*.*^41^, who explained it by co-integration into insertion site of extracellular DNA fragments derived from other cells lysed during electroporation. None of our discovered insertion sites could be associated to the chlorophyll biosynthetic pathway, thus though still incomplete, our insertion mapping implies that the expression cassette was randomly integrated into genome. Therefore, it is rather unlikely that the reduction of chlorophyll content in DARK-PALE lines was caused by disruption of chlorophyll biosynthetic genes.

### Influence of light on ketocarotenoid- and chlorophyll-biosynthesis in DARK-PALE transformants

**Figure 5:**
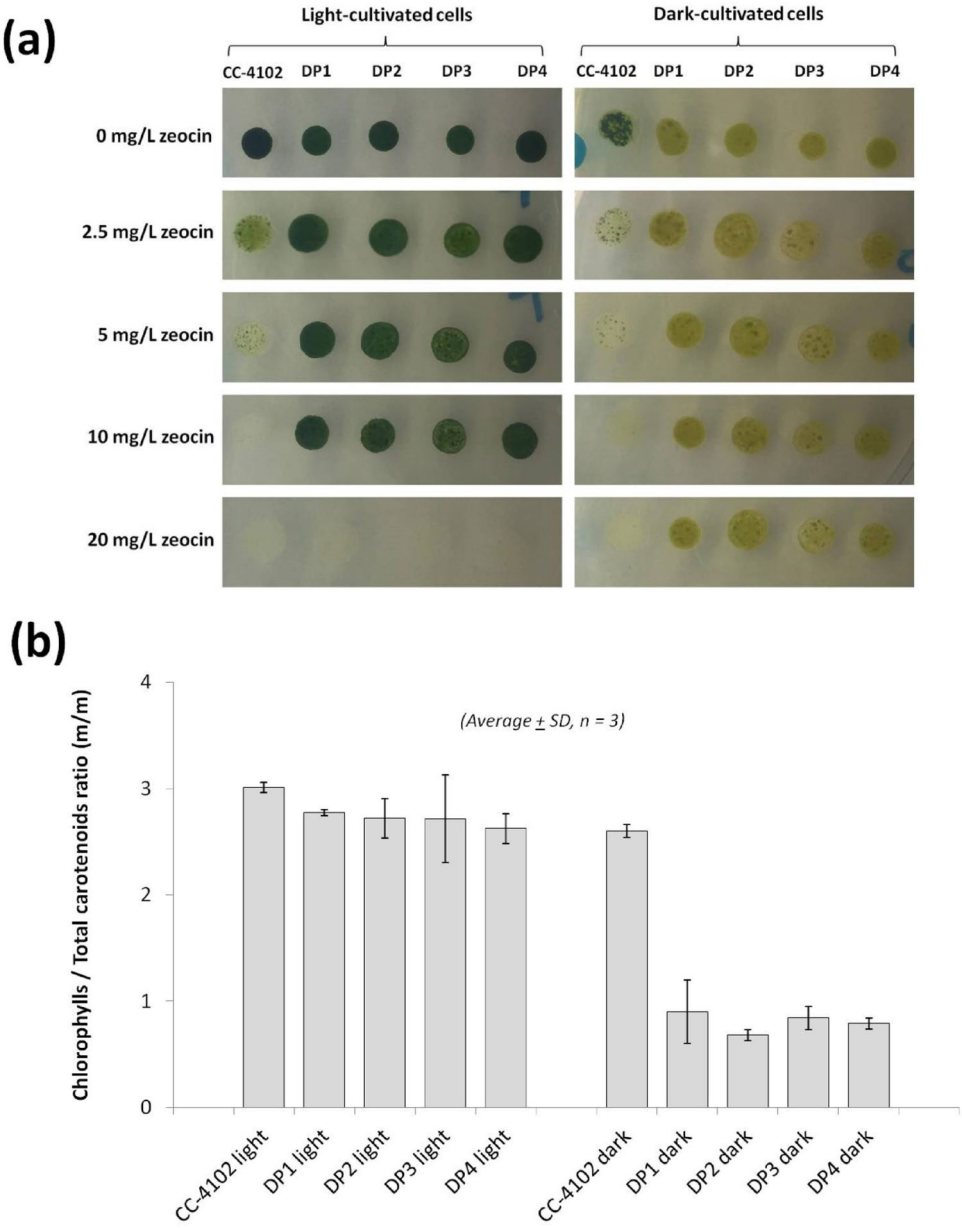
Changes of pigment profiles of DARK-PALE cells under light and dark conditions. (**a**) Light-cultivated DARK-PALE cells return to dark-green color and do not survive high zeocin concentration of 20 mg/L. (**b**) Comparison of chlorophylls/total carotenoids ratios of dark- and light-grown DARK-PALE cells. DP = DARK-PALE.

Initially we heterotrophically maintained DARK-PALE lines, i.e. in darkness on TAP-YP agar plates supplemented with zeocin (20 mg/L). We attempted to grow DARK-PALE cells mixotrophically on zeocin-containing TAP agar plates in the light but they repeatedly failed to grow. It turned out that the decrease of zeocin resistance was responsible for this failure. Light-cultivated DARK-PALE cells survived zeocin concentrations up to 10 mg/L but died completely at a concentration of 20 mg/L. Dark-grown DARK-PALE cells survived all tested concentrations (Fig. 5a). Intriguingly, light-grown DARK-PALE cells no longer showed the distinctive pale green color. Instead, they were dark green similar to non-transformed CC-4102 cells. HPLC analysis revealed the return of non-transformed chlorophyll/carotenoid ratio (Fig. 5b), as well as the absence of canthaxanthin in light-grown DARK-PALE cells (Fig. S5, Supplementary Material). The presence of an intact expression cassette was confirmed by PCR in both dark- and light-cultivated cells (Fig. S6, Supplementary Material).

## Discussion

Confirmation of the intact integration of the overexpression cassette’s intact integration into the genome by PCR resulted in very low positive rates: 10% among mixotrophic- and 12.5% among heterotrophic transformants. Such very low integration rates are unusual for the bicistronic ble2A system. Other publications have reported much higher rates of antibiotic-resistant lines harboring the gene of interest: 70%^42^ 93%^43^, 51.3%^44^. With plasmid pBR9 mCherry, mCherry-fluorescence was detected in 76% of zeocin-resistant colonies indicating that the incorporation rate of the mCherry gene into *Chlamydomonas* genome was high. PCR amplification for the *ble* gene resulted in positive results for all zeocin-resistant transformants (data not shown), data that confirm the presence of *ble* gene required for zeocin resistance. We postulate that in all PCR-negative lines, the expression cassette was fragmented and only the *ble* gene was incorporated into the genome. According to the transgene integration model proposed by Zhang et al.^40^, extracellular DNA is subjected to digestion by sequence-specific endonucleases before and during entry into recipient cells subjected to transformation. Cassette fragmentation and the subsequent low co-integration rates could be caused by sequence-specific cleavage of the CrBKT gene by such endonucleases.

In this approach, ketocarotenoid production in *Chlamydomonas reinhardtii* was associated with a decrease in chlorophyll content. This report is not the first time that such a correlation has been observed. Higher plants engineered to produce ketocarotenoids have much lower leaf chlorophyll contents compared to their non-transformed controls^45–50^. In microalgae such as *Haematococcus pluvialis*, *Chromochloris zofingiensis* and *Chlamydomonas reinhardtii*, ketocarotenoid biosynthesis is also accompanied by degradation of chlorophyll^28,51,52^. The mechanism behind this correlation is not well understood. However, its understanding is important to improve ketocarotenoid production in photosynthetic organisms. In higher plants, interference to chlorophyll biosynthesis could be avoided by limiting ketocarotenoid biosynthesis to non-photosynthetic tissues such as flowers or roots. Obviously, such options are unavailable in microalgae.

In CC-4102 cells, the predominant carotenoid is zeaxanthin, followed by ß-carotene. CrBKT can convert zeaxanthin to astaxanthin and ß-carotene to canthaxanthin with high efficiency^12^. Remarkably, we only detected canthaxanthin. We hypothesize that substrate and enzyme spatial separation is responsible for the absence of astaxanthin rather than low enzymatic activity. One of the first ketocarotenoid metabolic engineering attempts in *Chlamydomonas* was carried out by Leon et al.^18^, in which the authors overproduced HpBKT from *Haematococcus pluvialis* but could detect only 4-ketolutein, ketolation product from lutein, rather than astaxanthin and canthaxanthin. The authors attributed this finding to the enzyme’s inaccessibility to its substrate ß-carotene. In a more recent publication, Perozeni et al.^20^ also attempted to overexpress CrBKT in *Chlamydomonas npq2* mutant, but their study differs from ours. They directed CrBKT to the thylakoid membrane via fusion of the *psaD* chloroplast transit peptide (cTP^53^) to its N-terminus. As a result, both astaxanthin and canthaxanthin are detected in their psaD-CrBKT transformants. In our study, we intentionally did not include cTP because there is evidence of an intrinsic cTP in CrBKT sequence, both from *in silico* sequence analysis with PredAlgo^54^ as well as fluorescence translocation assay^20^. The lack of astaxanthin leads to the assumption that CrBKT was expressed and imported into chloroplast but not into thylakoid membrane, thus leaving it unable to metabolize zeaxanthin. Further studies will be needed to elucidate the exact location of CrBKT in *Chlamydomonas* chloroplast.

DARK-PALE transformants reverted back to dark green color when they were grown under light and lost ketocarotenoid production. This phenomenon persisted even when TAP-YP agar was used instead of TAP, indicating that a difference in medium compositions was not the reason for this phenomenon. Furthermore, promoter activity did not underline this difference because the HSP70/RBCS2 promoter is active under both light and dark conditions^55^. Loss of the transgene via genomic instability can also be excluded. Insertion-mediated disruption of the light-independent protochlorophyllide reductase genes (the so-called “yellow-in-dark” mutations) causes similar phenotypes^56^ but this explanation is inconsistent with our insertion mapping results. We postulate that interference of CrBKT overexpression with chlorophyll biosynthesis is likely the reason for the observed phenotypes. This phenomenon could explain why no ketocarotenoid-producing transformants were recovered from mixotrophic plates, even though there are more colonies on them. It is entirely possible that several transformant lines on mixotrophic plates were indeed capable of producing ketocarotenoids under the right conditions (dark, heterotrophic medium). However, being cultivated under the “wrong” conditions (light, mixotrophic medium), they failed to display the desired phenotype and were subsequently rejected during screening.

In this study, we showed that it is indeed possible to engineer ketocarotenoid pathway into *Chlamydomonas* green vegetative cells for production of the valuable pigment canthaxanthin. Despite this success, the usefulness of our transformants as a ketocarotenoid production platform is still limited by a number of factors including low ketocarotenoid concentration, interference to chlorophyll biosynthesis and the loss of phenotypes under light. In the bigger picture, these difficulties represent the common problems usually encountered by algal metabolic engineering and underscore the fact that our understanding of these organisms, despites three quarters century of intensive research, is still limited.

## Materials and methods

### Microorganisms and cultivation conditions

*Chlamydomonas* strain CC-4102 (genotype *npq2-2 npq1 lor1 arg7 mt* +) was obtained from the Chlamydomonas Resource Center, University of Minnesota USA. Algal cells were cultured mixotrophically in liquid or agar solidified Tris–Acetate-Phosphate (TAP) medium^57^ supplemented with 200 µg/mL l-arginine and at 25 °C under cool white daylight from fluorescent lamps (60 µE/m^2^ s); or heterotrophically in TAP medium supplemented with 0.3% peptone and 0.2% yeast extract (TAP-YP medium^34^) in the dark. For vector construction, *Escherichia coli* strain DH5α was cultivated at 37 °C in standard LB medium. For selection of algal and bacterial cells, zeocin (InvivoGen) and ampicillin (Carl-Roth) were added to final concentrations of 20 and 100 µg/mL respectively.

### Construction of CrBKT-overexpression vector

The plasmid pChlamy4 has been generated and distributed by Thermo Fisher as a vector optimized for protein expression in *Chlamydomonas reinhardtii*. The plasmid also yields higher levels of transgene expression and improved transgene stability by utilizing the bicistronic strategy, in which the selection marker, *ble* zeocin resistance gene from *Streptoalloteichus hindustanus*, is linked directly to the gene-of-interest’s sequence via the foot-and-mouth disease virus (FMDV) 2A self-cleaving sequence^42^. Gene expression is driven by the strong constitutive HSP70/RBCS2 promoter^55^. For subsequent protein detection and purification, the plasmid also harbors dual protein tags, namely 6xHis and V5.

We chose a truncated CrBKT in which a 116 amino acid-long sequence at its C-terminus was removed without affecting the protein’s activity (GenBank: AEA35045.1^12^) for overexpression in *Chlamydomonas*. The truncated CrBKT coding sequence was kindly donated by Dr. Jürgen Breitenbach, University Frankfurt, amplified via polymerase chain reaction (PCR) using primers 1502 and 1534 (Table 2) and cloned between the *Xho*I and *Xba*I sites of plasmid pChlamy4 (Thermo Fisher). Due to the presence of an *Xba*I site within the CrBKT sequence, the PCR products were instead digested with *Bcl*I, which created cohesive end compatible to *Xba*I-overhang. The resulting plasmid harbors the bicistronic construct of *ble* and *CrBKT* linked via the FMDV 2A sequence. At the C-terminus of CrBKT, there is the dual epitope V5-6xHis (Fig. 2).

**Table 2:** List of primers.

Primer	5′-3′ sequence	Description
1502	AATA CTCGAG ATG GGC CCT GGG ATA CAA CC	Used for amplification of CrBKT
1534	AAAA TCTAGA GA CGC CAG GGC TGC GCC	Used for amplification of CrBKT
1563	GCC ATA TGC ATG GCC ATC	5′-end mapping primer
1564	CGC ACC AAT CAT GTC AAG CCT CAG CG	5′-end mapping nested primer
1565	CTG GGC CTG GAC AGC ACC	3′-end mapping primer
1566	GGC GGG CTG GGC GTA TTT GAA GCG	3′-end mapping nested primer
1147	GTA ATA CGA CTC ACT ATA GGG C	Adaptor primer
1148	TGG TCG ACG GCC CGG GCT GG	Adaptor nested primer
1479	GAC CAG GTG GTG CCG GAC AAC ACC	Screening primer
1480	TTG CTC TCC ACG TCG CCC GCC AGC TTC	Screening primer
1491	AAAA GTCGAC CGC CAG GGC TGC GCC GCG	Screening primer

### Nuclear transformation of *Chlamydomonas*

Nuclear transformation of *Chlamydomonas* was attained with electroporation using modified protocol of Shimogawara et al*.*^58^. Briefly, *Chlamydomonas* was grown mixotrophically in TAP liquid medium supplemented with 200 mg/L l-arginine. Algal cells in early *log* phase (cell density 1–3 × 10^6^ cells/mL) were harvested, washed and concentrated to 3 × 10^8^ cells/mL in GeneArt MAX Efficiency Transformation Reagent for Algae (Thermo Fisher) following the manufacturer’s instructions. The cells were then dispersed into 250 µL aliquots. Five µg of *Sca*I-linearized plasmid was subsequently added and the mixture was chilled at 4 °C for 5 min. Electroporation was conducted with BioRad Gene Pulser system with 0.4 cm-gap cuvettes (BioRad). We chose the exponential decay mode with parameters set at 500 V, 50µF capacitance and 800 Ω resistance, which typically yielded a time constant around 50 ms. After electroporation, the cells were allowed to recover at room temperature for 15 min before being transferred to 10 mL fresh growth media supplemented with 40 mM sucrose, shaken overnight and plated on selection plates containing 20 mg/L zeocin the following day. Half of the cells were recovered and selected mixotrophically in TAP-arginine medium under 20 µE/m^2^ s white light while the other half were heterotrophically recovered in TAP-YP medium in the dark. Colonies appeared on agar plates after 2–3 weeks. We also established the following controls. For negative controls, cells were transformed without DNA and then recovered and screened under either mixotrophic or heterotrophic conditions. For the positive control, cells were transformed with 5 µg of linearized pBR9 mCherry plasmid^39^, then recovered and screened under mixotrophic conditions.

### Isolation of *Chlamydomonas* genomic DNA and PCR

Genomic DNA from *Chlamydomonas* was extracted using a modified cetyl trimethyl ammonium bromide (CTAB) method^59^. Briefly, cells were either harvested from 2–5 mL of densely grown liquid culture or scraped (a spatula full) from agar plates, washed with double distilled water (ddH_2_O) and resuspended in 500 µL lysis buffer containing 2% w/v CTAB, 100 mM Tris–HCl (pH 8), 1.4 M NaCl, 20 mM ethylenediaminetetraacetic acid (EDTA) and 2% v/v freshly added ß-mercaptoethanol. The samples were incubated at 65 °C for 1 h and extracted with a standard phenol–chloroform extraction protocol. DNA was precipitated from the aqueous phase with 0.7 volume of isopropanol. The DNA pellet was washed once with 70% ethanol, air-dried and dissolved in 20 µL ddH_2_O. To remove RNA and reconstitute DNA, the samples were treated with RNase A (1 mg/mL) overnight at room temperature.

We performed PCR with Biotherm Taq polymerase (Genecraft) and 10–50 ng of extracted genomic DNA. Due to the high GC content of *Chlamydomonas* genome, we added dimethyl sulfoxide (DMSO) to the reaction mixture to final concentration of 5% v/v.

### Pigment extraction and analysis

We extracted pigments from *Chlamydomonas* with 100% methanol. We measured the sample’s absorption at 470, 652 and 665 nm with a WPA Biowave S2100 UV/Vis Diode Array Spectrophotometer. The total chlorophylls and carotenoid concentrations were estimated with the following equations^60^.

$$ \begin{aligned} & {\text{c}}[Chlorophyll\,a]\,\left( {\upmu {\text{g/mL}}} \right) = {16}.{72} \times {\text{A}}_{{{665}}} {-}{ 9}.{16} \times {\text{A}}_{{{652}}} \\ & {\text{c}}[Chlorophyll\,b]\,\left( {\upmu {\text{g/mL}}} \right) = {34}.0{9} \times {\text{A}}_{{{652}}} {-}{ 15}.{28} \times {\text{A}}_{{{665}}} \\ & {\text{c}}[Total\,carotenoids]\,\left( {\mu {\text{g}}/{\text{mL}}} \right) = \frac{{1000{ } \times {\text{A}}470 - {\text{c}}\left[ { Chlorophyll\, a } \right] \times { }1.63 - {\text{c}}\left[ { Chlorophyll\, b } \right] \times { }104.96}}{221} \\ \end{aligned} $$

For high performance liquid chromatography (HPLC) analysis, we sequentially extracted pigments with 200 µL methanol, 200 µL acetone and 200 µL chloroform. We combined the organic extracts and added 1,000 mL dH_2_O. The lower phase which contained chlorophylls and carotenoids was collected, dried under a stream of dry nitrogen gas and finally dissolved in methanol. We separated pigments on a C30 YMC-carotenoid column (YMC, Japan, 250 mm × 4.6 mm, 5 µm particles) using a published protocol^61^. The mobile phases contained Solvent A: 81% (v/v) methanol, 15% methyl *tert*-butyl ether (MTBE), 4% H_2_O; and Solvent B: 8% methanol, 88% MTBE, 4% H_2_O. We applied the following gradient program: 0–30 min: 0–67% Solvent B; 30–35 min: 100% Solvent B; 35–40 min: 0% Solvent B. We detected pigments with a variable wavelength detector (VWD) model G1314A (Agilent) set at 450 nm. Absorption spectra from 280 to 640 nm were recorded with diode array detector (DAD) model G4212B (Agilent).

### Mapping of transgene insertion sites via genome walking

We mapped the insertion sites of transformants with the genome walking method described by Siebert et al*.*^62^ and Pollock et al*.*^41^. Briefly, genomic DNA was extracted from *Chlamydomonas* transformants and digested overnight at 37 °C by a mixture of three blunt-end cutting restriction enzymes: *Eco*RV, *Nru*I and *Pvu*II. We prepared a blunt-ended adaptor from two oligonucleotides: one 8 nucleotides (nt)-long (oligo 389) and the other 48 nt long (oligo 390; Table 2). Prior to annealing, oligo 389 was phosphorylated for 2 h at 37 °C by T4 polynucleotide kinase (NEB) in 1 × T4 ligase buffer supplemented with 1 mM ATP. We mixed 500 pmol of phosphorylated oligo 389 and 1,000 pmol of oligo 390 in 1 × Green buffer (Thermo Fisher) to a final volume of 40 µL, heated to 95 °C for 5 min and slowly cooled down to 4 °C (a rate of 1.5 °C/min) in an ordinary thermocycler. In the following step, we ligated the adaptor to the blunt end of DNA fragments at 16 °C overnight. The ligation mixture comprised 40 ng digested genomic DNA, 4 µL adaptor, 1 mM ATP, 8 units of T4 ligase (Thermo Fisher) and 1 × T4 ligase buffer. After the reaction was stopped by heating at 65 °C for 20 min, we diluted the ligation mixture 1:10 with ddH_2_O and used it as the template for PCR.

For amplification of the upstream- and downstream flanking sequences of the transgene, we performed two rounds of PCR. Using adaptor-ligated genomic DNA fragments as template, the first round of PCR utilized an adaptor primer (primer 1147) and a gene-specific primer (primer 1563 or 1565). In the second “nested” PCR, we diluted PCR products from the first round 1:50 with ddH_2_O and amplified them using adaptor primer 1148 and gene-specific primers 1564 and 1566. The positions of these primers are shown in Fig. 2. Both PCRs utilized touch-down programs to minimize nonspecific amplification (Fig. S1) with high-processivity Taq polymerase (Genaxxon). We cloned nested PCR products in the *Eco*RV site of pBluescript II SK( +) via TA cloning^63^ and sent them for sequencing at Mycrosynth Seqlab (Göttingen, Germany) using the M13 Forward (-20) primer (TGT AAA ACG ACG GCC AG). We then compared sequencing data to the *Chlamydomonas* genome database on Phytozome (https://phytozome.jgi.doe.gov).

### Statistical analysis

We determined statistical significance using Student’s t-test, namely the "Two-Sample Assuming Equal Variances with Analysis ToolPak Add-in" of Microsoft Office Excel 2007.

### Ethical approval

 This article does not contain any studies with human participants or animals performed by any of the authors.

## Supplementary information

Supplementary information
