# Supplementary material for: Metabolic engineering of ketocarotenoids biosynthetic pathway in Chlamydomonas reinhardtii strain CC-4102
Source: Sci Rep. 2020 Jul 1;10:10688. doi: 10.1038/s41598-020-67756-2 (PMC7329852; doi:10.1038/s41598-020-67756-2)
Supplement: Supplementary file 1 — Supplementary information [file 41598_2020_67756_MOESM1_ESM.pdf]

SUPPLEMENTARY MATERIAL

Metabolic Engineering of Ketocarotenoids Biosynthetic Pathway in *Chlamydomonas reinhardtii* strain CC-4102

Nam Trung Tran[1*], Ralf Kaldenhoff[1]

[1]Department of Biology, Applied Plant Sciences, Technische Universität Darmstadt, Schnittspahn Strasse 10, D-64287, Darmstadt, Germany

*Corresponding author: Tel: +49 6151 16-22255, E-mail address: tran@bio.tu-darmstadt.de

Both upstream and downstream regions of insertion sites in all four DARK-PALE lines were mapped via genome walking. Tochdown PCR protocols are shown in **Fig. S1**. PCR mapping showed bands whose sizes ranged from 200 to 1000 bp (**Fig. S2**). The largest bands were cut from gel, cloned into pBluescript plasmid and sequenced via Sanger sequencing. Results are summarized in **Table S1**. Unfortunately, we were unable to pinpoint the insertion sites in DARK-PALE 3 and DARK-PALE 4. In their 5'-end mapping, only sequences from transformation plasmid were obtained, implying that the insertion sites happened to be the same as mapping's restriction sites. 3'-end mapping yielded sequences of non-*Chlamydomonas* origins. BLAST analysis indicated that these sequences were 87% identical to *Microbacterium* sp. DNA, hinting at bacterial contamination. Equally, 5'-end mapping of DARK-PALE 2 was also unsuccessful since insertion site is only four nucleotides away from restriction site.

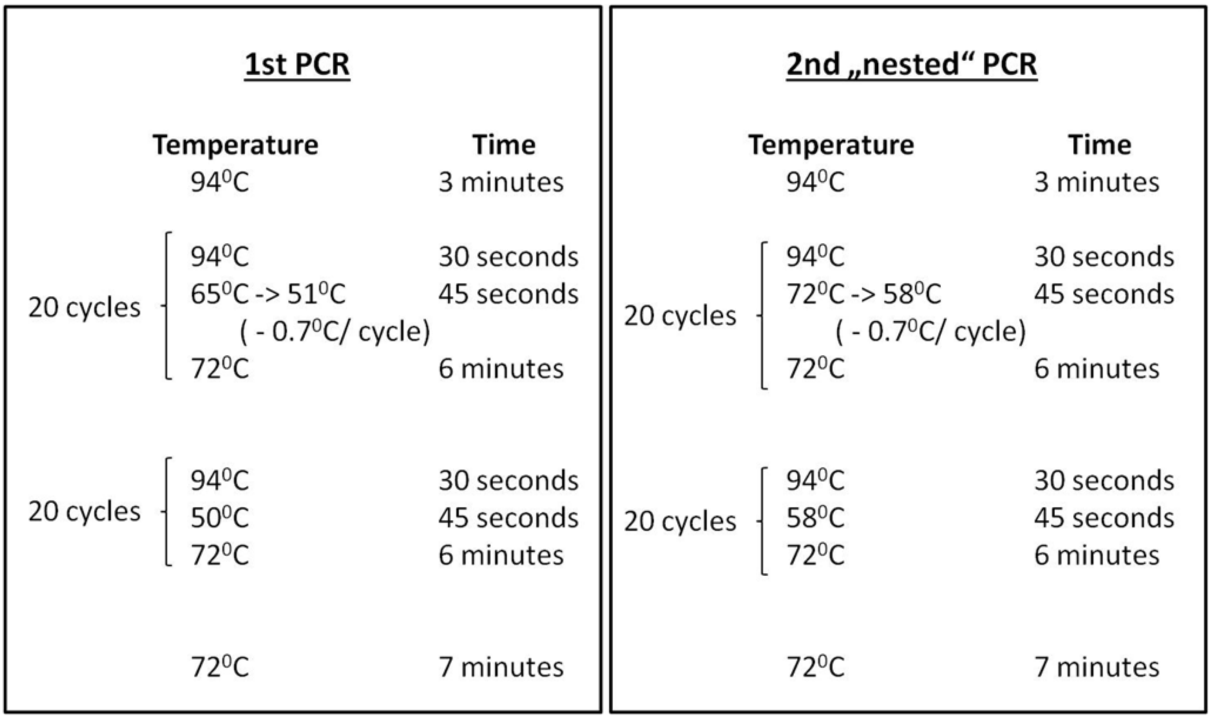

Fig. S1 Tochdown PCR protocol for insertion mapping

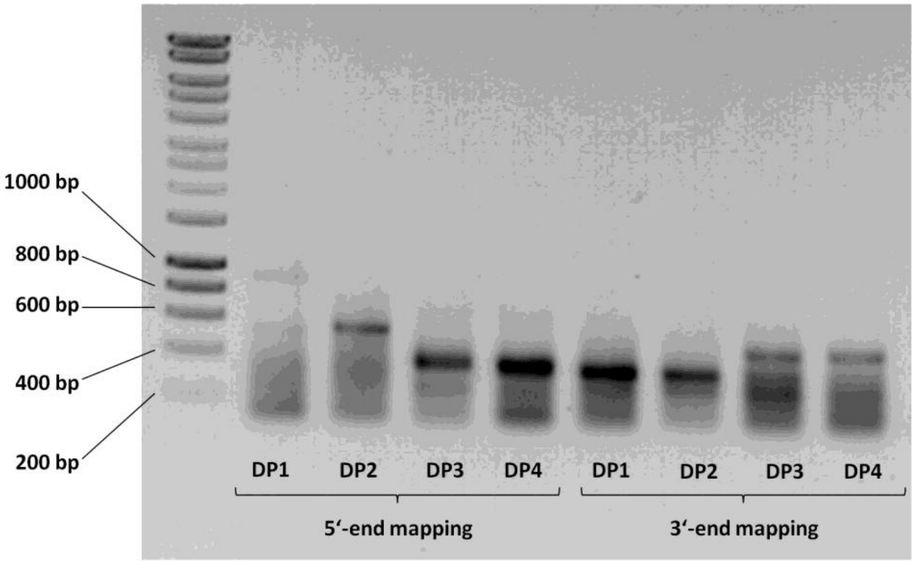

Fig. S2 Nested PCR for Insertion mapping of both 5'- and 3-ends of all four DARK-PALE lines via genome walking. Biggest bands were excised from gel, cloned into pBluescript via TA cloning and sequenced. DP = DARK-PALE

Table S1: Insertion mapping of DARK-PALE transformants

		**Obtained sequence**	**Positions/ annotations**
DARK-PALE1	5'-end	0.8 kb	Chromosome 12:2721079..2721880 (intron sequence)
	3'-end	0.3 kb	Chromosome 14:2789910..2790198 (Unconventional Myosin-XIX)
DARK-PALE2	5'-end	0.55 kb	Unsuccessful, only 4 nucleotides from *Chlamydomonas* genome is identified, the rest are from plasmid
	3'-end	0.3 kb	Chromosome 12:4680191..4680470 Oligosaccharyltransferase, alpha subunit (ribophorin I)
DARK-PALE3	5'-end	0.3 kb	Unsuccessful, only plasmid sequence is obtained
	3'-end	0.3 kb	Contaminating sequence
DARK-PALE4	5'-end	0.45 kb	Unsuccessful, only plasmid sequence is obtained
	3'-end	0.45 kb	Contaminating sequence

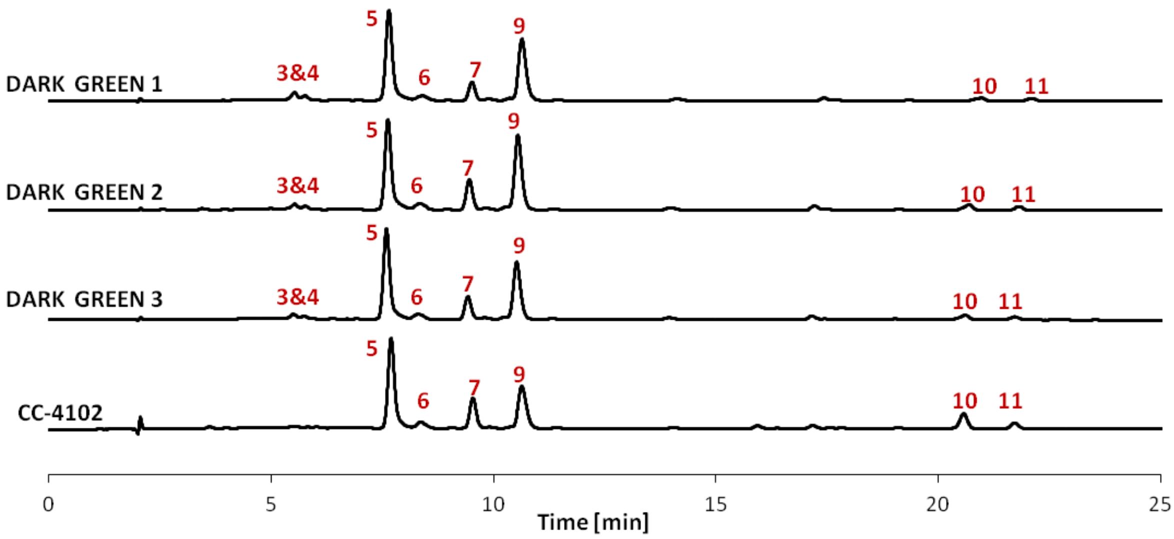

	Chlorophyll a [pg/cell]	Chlorophyll b [pg/cell]	Zeaxanthin [pg/cell]	ß-carotene [pg/cell]
DARK GREEN 1	1.54	0.34	0.54	0.12
DARK GREEN 2	1.75	0.23	0.67	0.13
DARK GREEN 3	1.65	0.30	0.54	0.14
CC-4102	1.88	0.26	0.60	0.22

Fig. S3: Pigment profiles of three DARK GREEN transformants compared to non-transformed CC-4102 cells. All cells were grown in dark on heterotrophic medium (TAP-YP). Peaks identification: (3), (4): putative chlorophyll degradation products, (5): chlorophyll *b*, (6): lutein, (7): zeaxanthin, (9): chlorophyll *a*, (10) and (11): ß-carotene. Estimations of pigment concentrations are shown in the table. Chlorophyll a & b's concentration were measured spectrophotometrically using method of Lichtenthaler 2001. Zeaxanthin and ß-carotene concentrations were estimated from comparing their peak areas with their corresponding standards. Cell numbers were obtained with Countess II Automated Cell Counter (Thermo Fisher)

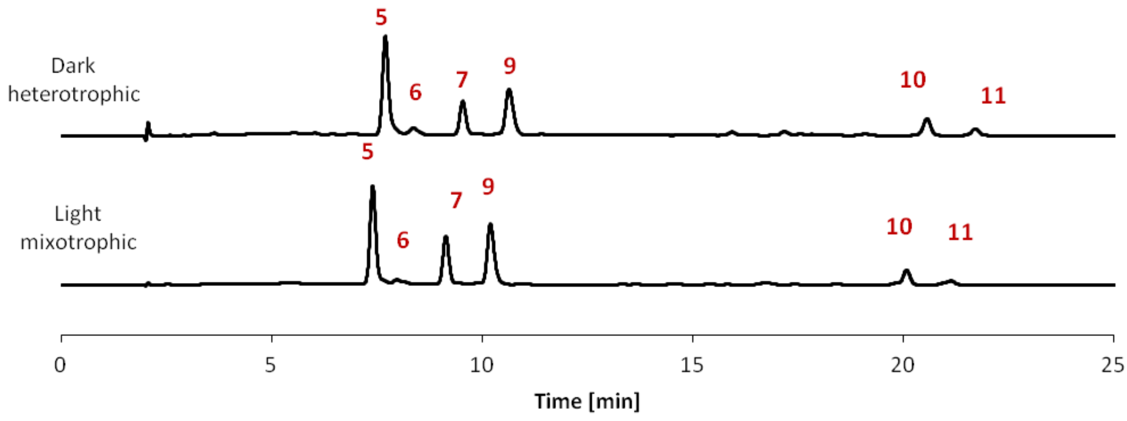

	Chlorophyll a [pg/cell]	Chlorophyll b [pg/cell]	Zeaxanthin [pg/cell]	ß-carotene [pg/cell]
Dark heterotrophic	1.88	0.26	0.60	0.22
Light mixotrophic	2.03	0.40	0.66	0.15

Fig. S4: Pigment profile comparison of non-transformed CC-4102 cells grown in dark on heterotrophic medium (TAP-YP) as well as in light on mixotrophic medium (TAP-arginine). Peaks identification: (5): chlorophyll *b*, (6): lutein, (7): zeaxanthin, (9): chlorophyll *a*, (10) and (11): ß-carotene. Estimations of pigment concentrations are shown in the table. Chlorophyll a & b's concentration were measured spectrophotometrically using method of Lichtenthaler 2001. Zeaxanthin and ß-carotene concentrations were estimated from comparing their peak areas with their corresponding standards. Cell numbers were obtained with Countess II Automated Cell Counter (Thermo Fisher)

Table S2: Estimations of pigment concentrations of DARK GREEN 1, DARK PALE 1 and non-transformed CC-4102 cells grown under the same conditions (in dark on heterotrophic medium). Chlorophyll a & b's concentration were measured spectrophotometrically using method of Lichtenthaler 2001. Zeaxanthin and ß-carotene concentrations were estimated from comparing their peak areas with their corresponding standards. Cell numbers were obtained with Countess II Automated Cell Counter (Thermo Fisher)

	**Chlorophyll a [pg/cell]**	**Chlorophyll b [pg/cell]**	**Zeaxanthin [pg/cell]**	**ß-carotene [pg/cell]**	**Canthaxanthin [pg/cell]**
DARK GREEN 1	1.54	0.34	0.54	0.12	not detected
DARK PALE 1	0.66	0.06	0.75	0.16	0.082
CC-4102	1.88	0.26	0.60	0.22	not detected

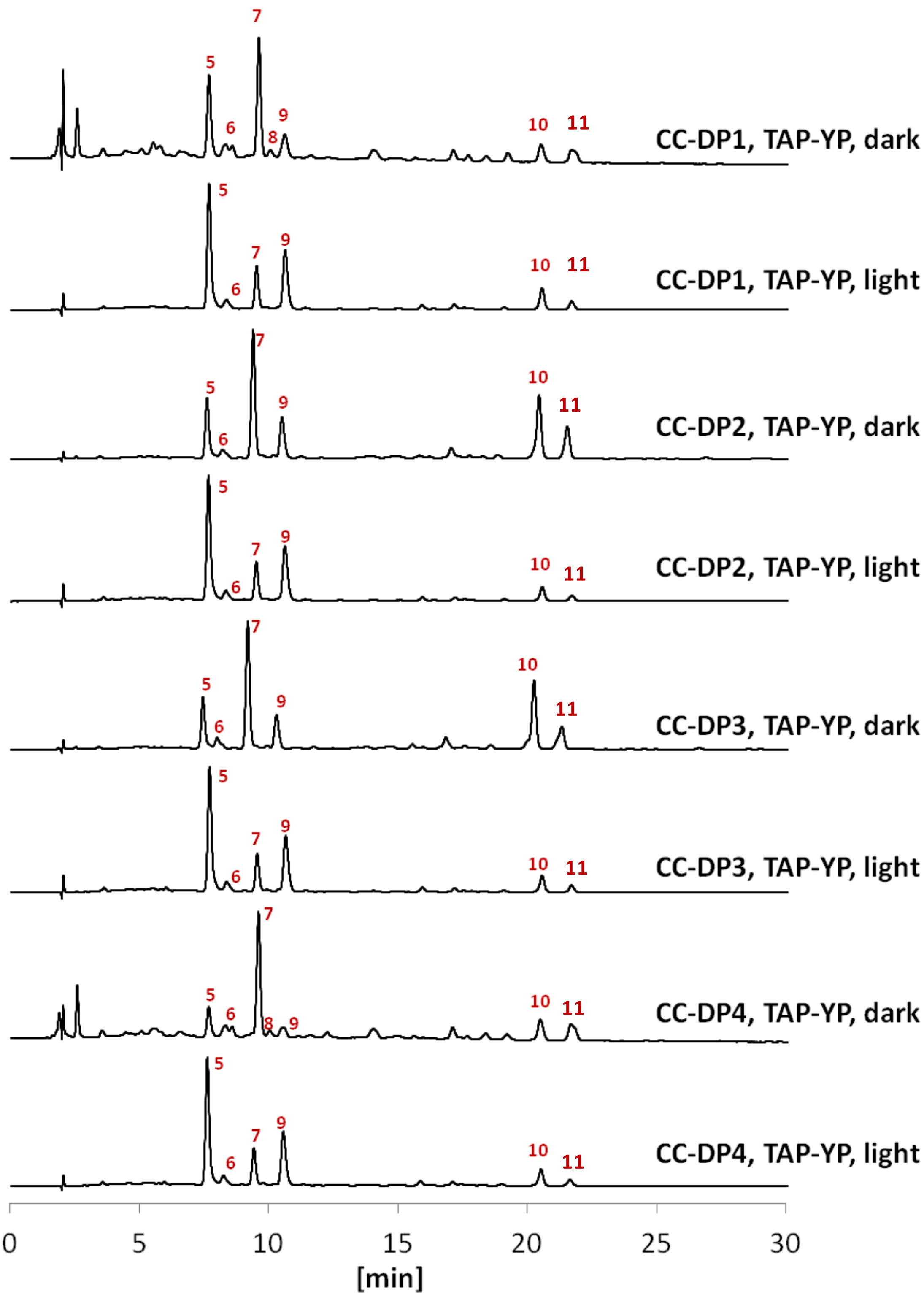

Fig. S5: When DARK-PALE *Chlamydomonas* cells (CC-DP) were cultivated in light, their pigment profile changes dramatically. HPLC analysis revealed the return of chlorophylls, which reverted cell's color from pale green to dark green. Also canthaxanthin was no longer detectable. Pigments: 5 = chlorophyll b, 6 = lutein, 7 = zeaxanthin, 8 = canthaxanthin, 9 = chlorophyll a, 10 & 11 = α- and ß-carotene. Detection at 450nm.

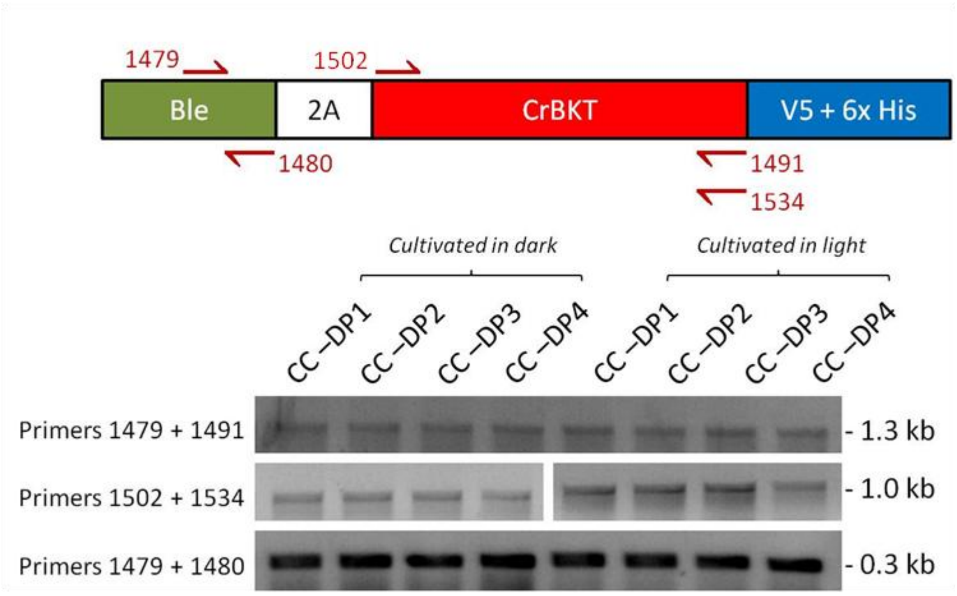

Fig. S6: The Ble2A-CrBKT was still in DARK-PALE cell's genome regardless if they were cultivated in dark or in light. Three primers pairs were used to confirm the transgene: 1479 + 1491 (Ble2A-CrBKT gene), 1502 + 1534 (CrBKT gene) and 1479 + 1480 (Ble gene). Expected amplicon sizes were 1.3 kb, 1.0 kb and 0.3 kb respectively. Primer binding positions were also shown. CC-DP 1-4 are DARK PALE 1-4.

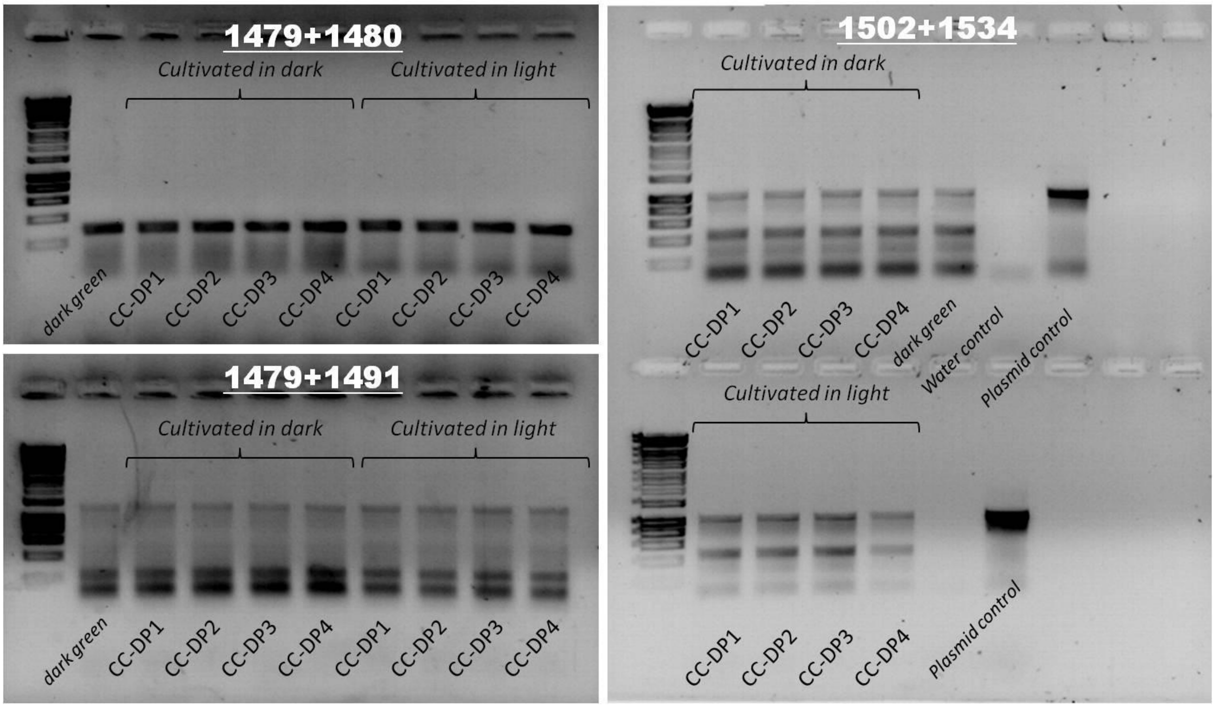

Fig. S7: Full-length gels of PCR results shown in **Fig. S6**. *Upper left*: PCR with 1479 and 1480 primers (Ble gene). *Lower left*: PCR with 1479 and 1491 primers (Ble2A-CrBKT gene). *Right*: PCR with 1502 and 1534 primers (CrBKT gene). *Dark green*: PCR with extracted gDNA from a positive dark green transformant. Water control: PCR with ddH_2O instead of gDNA template. Plasmid control: PCR with plasmid (pChlamy4 CrBKT V5H) instead of gDNA template. DNA ladder was Hyperladder I (Bioline).
